# Systematic dissection of the genomic mechanisms underlying differential tauopathy risks associated with the human 17q21.31 locus

**DOI:** 10.1002/alz.089768

**Published:** 2025-01-03

**Authors:** Le Qi, Chiara Pedicone, Kathryn Bowles, Jiani Yin, Alison M. Goate, Daniel H. Geschwind

**Affiliations:** ^1^ University of California, Los Angeles School of Medicine, Los Angeles, CA USA; ^2^ Icahn School of Medicine at Mount Sinai, New York, NY USA; ^3^ University of Edinburgh, Edinburgh, Scotland United Kingdom

## Abstract

**Background:**

The genetic association between 17q21.31 and increased risk for tauopathies, such as Progressive Supranuclear Palsy, is well established. However, the mechanisms driving the differential disease risks between two major haplotypes, H1 and H2 are unclear. This is mainly due to a 970KB inversion between H1 and H2 haplotypes, leading to a 1.5Mb region where strong LD confounds the identification of causal variants, as well as our understanding of the gene regulatory mechanisms contributing to disease.

**Method:**

To identify genetic and epigenetic changes between the H1 and H2 haplotypes that drive neurodegeneration, we obtained homozygous H1/H1 and H2/H2 iPSCs from healthy donors with European ancestry. We performed comprehensive genomic profiling, including RNA‐seq, ATAC‐seq and Hi‐C on neurons, astrocytes and microglia independently derived from iPSCs. Differentially expressed genes, accessible chromatin regions (DARs) and chromatin interactions were analyzed between H1/H1 and H2/H2 cells. Integration of different modalities was done to uncover causal H1/H2‐associated variants and their effects on gene regulatory mechanisms.

**Result:**

We identified 6682 and 8603 genome‐wide DARs, as well as 5 and 27 DARs at the 17q21.31 locus in H1/H1 and H2/H2 neurons respectively. In H2 neurons, genes with reduced accessibility relative to H1 neurons, in either promoters or distal enhancers, showed enrichment in Gene Ontology terms associated with axon development and synaptic signaling; genes with increased relative accessibility in H2 neurons were associated with cell cycle and immune responses. Transcription factor (TF) binding motif analysis showed that neuronal differentiation‐related TFs were enriched in DARs reduced in H2 neurons. We detected reduced accessibility in *MAPT* promoters in H2 neurons, consistent with previous findings. We identified several H1/H2 haplotype‐divergent SNPs within the *MAPT* promoter peak, that disrupt binding motifs for N‐MYC, CTCF, SP2 and AP‐2.

**Conclusion:**

Through analysis of chromatin accessibility, we pinpointed several pathways involved in neuronal signaling that differentiate neurons from H1/H1 and H2/H2 individuals, including DARs at the *MAPT* locus that likely account for reduced expression on H2 haplotypes. We also identified several haplotype‐divergent SNPs predicted to interrupt TF binding required for *MAPT* expression. Both of these changes potentially contribute to reduced risk for tauopathy in H2/H2 individuals.

